# Supplementary material for: Scywalker: scalable end-to-end data analysis workflow for long-read single-cell transcriptome sequencing
Source: Bioinformatics. 2024 Sep 10;40(9):btae549. doi: 10.1093/bioinformatics/btae549 (PMC11419950; doi:10.1093/bioinformatics/btae549)
Supplement: btae549_Supplementary_Data [file btae549_supplementary_data.pdf]

# Supplementary Material

## Code

Code isoquant run: How isoquant is run internally

```
isoquant3 \
    --data_type $data_type \
    --model_construction_strategy $model_construction_strategy \
    --splice_correction_strategy $splice_correction_strategy \
    --transcript_quantification $quantification \
    --gene_quantification $gene_quantification \
    --threads 1 \
    --reference $refseq \
    --bam $tempbam \
    --keep_tmp \
    --genedb $tempgenedb \
    -o $regdir.temp

where
      refseq = the genome reference sequence
      tempbam = a temporary bam file containing only reads from the region to be
      analyzed
      tempgenedb = a temporary isoquant genedb file containing only transcripts
      from the region to be analyzed
      regdir = (temporary) ouput directory for the current region

with under default settings for ONT data:
      data_type = nanopore
      model_construction_strategy = default_ont
      splice_correction_strategy = default_ont

And under default settings for PacBio data
      data_type = pacbio
      model_construction_strategy = default_pacbio
      splice_correction_strategy = default_pacbio

# The gene_quantification and transcript_quantification are not relevant,
# as these results at this level are not used: # The counts in the results
# are calculated by scywalker at the end after correcting for the analysis
# in separate regions, and for different settings at the same time (not only
# all)
```

Code code_comparison: code used to run scywalker, BLAZE-FLAMES, and wf-single-cell on the scmixology2 data set

```
# scmixology2_ont scywalker benchmark run
# =======================================
```

```
cd /tmp/benchmark-scmixology2
/usr/bin/time -o time.txt -v \
    scywalker -stack 1 -v 2 -d 24 \
    -sc_expectedcells 183 \
    -cellmarkerfile ../../markers.tsv \
    -threads 4 \
    -refdir /complgen/refseq/hg38 \
    /tmp/benchmark-scmixology2/samples/scmixology2_ont \
    >& startup_process_sample.log

# scmixology2 wf-single-cell benchmark run
# ==========================================
cd ~/benchmarks/
mkdir -p /tmp/benchmark-scmixology2/samples/scmixology2_wf/FASTQ
cd /tmp/benchmark-scmixology2/samples/scmixology2_wf
zcat ../../FASTQ/*.FASTQ.gz | gzip > FASTQ/scmixology2_wf.FASTQ.gz

export NXF_SINGULARITY_CACHEDIR=/tmp/singularity

echo 'executor {
      $local {
      cpus = 24
      memory = "500 GB"
      }
}' > my-config.cfg

/usr/bin/time -o time.txt -v \
    nextflow run epi2me-labs/wf-single-cell \
    -r v0.2.8 \
    -profile singularity \
    -c my-config.cfg \
    -w wf-single-cell_workspace \
    --ref_genome_dir /complgen/bin/cellranger-7.1.0/refs/refdata-gex-GRCh38-2020-A \
    --FASTQ FASTQ \
    --sample scmixology2_wf \
    --kit_name 3prime \
    --kit_version v3 \
    --expected_cells 183 \
    --out_dir wf-single-cell \
    >& wf-single-cell.log

# scmixology2 BLAZE-FLAMES benchmark run
# ========================================
# install
# -------
cd /tmp/benchmark-scmixology2/samples/scmixology2_fm
export mambaversion=22.11.1-4
curl -L -O
"https://github.com/conda-forge/miniforge/releases/download/$mambaversion/Mambaforge
-$mambaversion-Linux-x86_64.sh"
unset PYTHONPATH
```

```
rm -rf /home/data/mambaforge
bash Mambaforge-$mambaversion-Linux-x86_64.sh -b

PATH=/home/data/mambaforge/bin:$PATH
mamba init bash
. ~/.bash_profile

wget https://github.com/shimlab/BLAZE/releases/download/v1.1.0/BLAZE_v1.1.0.zip
unzip BLAZE_v1.1.0.zip
cd BLAZE
mamba env remove -n blaze
conda config --set channel_priority false
mamba env create -f conda_env/environment.yml

mamba env remove -n flames
mamba create -y -n flames \
       python=3.7 samtools pysam minimap2 numpy editdistance \
       -c bioconda -c conda-forge
mamba activate flames
git clone https://github.com/LuyiTian/FLAMES.git
cd /tmp/benchmark-scmixology2/samples/scmixology2_fm/FLAMES/src
g++ -std=c++11 -lz -O2 -o match_cell_barcode ssw/ssw_cpp.cpp ssw/ssw.c
match_cell_barcode.cpp kseq.h edit_dist.cpp


# run
# ---
cd /tmp/benchmark-scmixology2/samples/scmixology2_fm
PATH=/home/data/mambaforge/bin:$PATH
mamba init bash
. ~/.bash_profile

#### BLAZE
mamba activate blaze
export
PATH=/home/data/mambaforge/envs/blaze/bin:/home/data/mambaforge/condabin:$PATH
/usr/bin/time -o time_blaze.txt -v \
    python BLAZE/bin/blaze.py --expect-cells=183 --kit-version=v3 --threads=24 \
    FASTQ
mamba deactivate

#### FLAMES
mamba activate flames
/usr/bin/time -o time_match_cell_barcode.txt -v \
    FLAMES/src/match_cell_barcode FASTQ barcode_statistics.tsv barcoded.FASTQ.gz
whitelist.csv 2 12

/usr/bin/time -o time_flames.txt -v \
       python FLAMES/python/sc_long_pipeline.py \
       --infq barcoded.FASTQ.gz \
       --outdir FLAMES_output \
```

```
       --genomefa
/complgen/bin/cellranger-7.1.0/refs/refdata-gex-GRCh38-2020-A/fasta/genome.fa \
       --gff3
/complgen/bin/cellranger-7.1.0/refs/refdata-gex-GRCh38-2020-A/genes/genes.gtf \
       --config_file FLAMES/python/config_sclr_nanopore_default.json

mamba deactivate

# brain1_ont scywalker benchmark run
# ====================================
cd /tmp/benchmark-brain1/samples/brain1_ont
/usr/bin/time -o time.txt -v \
       scywalker -stack 1 -v 2 -d 24 \
       -sc_expectedcells 15000 \
       -cellmarkerfile Cell_markers_Tijs_Jan2024.tsv \
       -threads 4 \
       -refdir /complgen/refseq/hg38 \
       /tmp/benchmark-brain1/samples/brain1_ont \
       >& startup_process_sample.log

# brain1_fm BLAZE-FLAMES benchmark run
# ====================================
cd /tmp/benchmark-brain1/samples/brain1_fm

#### BLAZE
mamba activate blaze
/usr/bin/time -o time_blaze.txt -v \
    python BLAZE/bin/blaze.py --expect-cells=15000 --kit-version=v3 --threads=24 \
    fastq
mamba deactivate

#### FLAMES
mamba activate flames
/usr/bin/time -o time_match_cell_barcode.txt -v \
       FLAMES/src/match_cell_barcode fastq barcode_statistics.tsv barcoded.fastq.gz
whitelist.csv 1 12

/usr/bin/time -o time_flames.txt -v \
       python FLAMES/python/sc_long_pipeline.py \
              --infq barcoded.fastq.gz \
              --outdir FLAMES_output \
              --genomefa
/complgen/bin/cellranger-7.1.0/refs/refdata-gex-GRCh38-2020-A/fasta/genome.fa \
              --gff3
/complgen/bin/cellranger-7.1.0/refs/refdata-gex-GRCh38-2020-A/genes/genes.gtf \
              --config_file FLAMES/python/config_sclr_nanopore_default.json

mamba deactivate

# Make comparison files
# —--------------------
src=FLAMES_output
sample=brain1_fm
sw csv2tsv $src/transcript_count.csv.gz \
    | sw keyvalue -idfields 'transcript_id gene_id' -keyname cell -valuename count \
    | sw select -f 'transcript=$transcript_id geneid=$gene_id cell count' \
    | sw zst \
```

```
    > sc_isoform_counts_filtered-$sample.tsv.zst
sw select -optimization memory -g 'geneid * cell *' -gc 'sum(count)'
sc_isoform_counts_filtered-$sample.tsv.zst \
    | sw select -f 'geneid cell count=$sum_count' \
    | sw zst \
    > sc_gene_counts_filtered-$sample.tsv.zst
sw select -g 'cell *' -gc 'sum(count)' sc_gene_counts_filtered-$sample.tsv.zst \
    | sw select -f 'cellbarcode=$cell count=$sum_count' \
    | sw zst \
    > umis_per_cell_filtered-$sample.tsv.zst
```

# Supplementary Tables

Supplementary Table 1: overview data sets used in this study

| sample | Number of reads | average read size | number of megabases | source |
|---|---|---|---|---|
| brain1 | 109,533,124 | 837.36 | 91,719.00 | |
| brain2 | 92,847,419 | 795.76 | 73,883.92 | |
| brain3 | 114,379,763 | 592.52 | 67,772.50 | |
| brain4 | 93,664,035 | 786.75 | 73,689.71 | |
| brain1_srs | 415,477,694 | 90.00 | 37,392.99 | |
| brain2_srs | 364,326,042 | 90.00 | 32,789.34 | |
| brain3_srs | 266,656,691 | 90.00 | 23,999.10 | |
| brain4_srs | 653,867,678 | 90.00 | 58,848.09 | |
| scmixology2 | 25,517,285 | 1,120.41 | 28,589.86 | SRR12282458 |
| scmixology2_srs | 107,473,860 | 91.00 | 9,780.12 | SRR12282457 |
| plant1 | 220,703,149 | 771.63 | 170,301.49 | |
| plant2 | 230,532,473 | 967.72 | 223,091.72 | |
| plant1_srs | 377,557,437 | 90.00 | 33,980.17 | |
| plant2_srs | 467,199,393 | 90.00 | 42,047.95 | |
| hg002_pacbio | 98,964,033* | 1,039.31 | 102,854.37 | (1) |
| hg002_srs | 262,936,766 | 100 | 26,294.68 | (2) |
| pbmc_pacbio | 43,788,133* | 737.59 | 32,297.80 | (3) |

(1) https://downloads.pacbcloud.com/public/dataset/MAS-Seq/DATA-Revio-Kinnex-HG002-10x5p/1-Sreads/segmented.bam
(2) https://downloads.pacbcloud.com/public/dataset/MAS-Seq/DATA-Revio-Kinnex-HG002-10x5p/Illumina
(3) https://downloads.pacbcloud.com/public/dataset/Kinnex-single-cell-RNA/DATA-MAS-SQ2-PBMC_10kcells/1-Sreads/segmented.bam

* Segmented reads

Supplementary Table 2 marker genes for human brain

| geneid | marker | celltype |
|---|---|---|
| ENSG00000067715.15 | SYT1 | Excitatory neurons |
| ENSG00000154146.13 | NRGN | Excitatory neurons |
| ENSG00000091664.9 | SLC17A6 | Excitatory neurons |
| ENSG00000104888.10 | SLC17A7 | Excitatory neurons |
| ENSG00000070808.17 | CAMK2A | Excitatory neurons |
| ENSG00000067715.15 | SYT1 | Inhibitory neurons |
| ENSG00000128683.14 | GAD1 | Inhibitory neurons |
| ENSG00000136750.13 | GAD2 | Inhibitory neurons |
| ENSG00000171885.18 | AQP4 | Astrocytes |
| ENSG00000110436.13 | SLC1A2 | Astrocytes |
| ENSG00000131095.14 | GFAP | Astrocytes |
| ENSG00000129244.9 | ATP1B2 | Astrocytes |
| ENSG00000080493.19 | SLC4A4 | Astrocytes |
| ENSG00000165795.25 | NDRG2 | Astrocytes |
| ENSG00000125398.8 | SOX9 | Astrocytes |
| ENSG00000182578.14 | CSF1R | Microglia |
| ENSG00000169896.18 | ITGAM | Microglia |
| ENSG00000169313.10 | P2RY12 | Microglia |
| ENSG00000019582.17 | CD74 | Microglia |
| ENSG00000168329.14 | CX3CR1 | Microglia |
| ENSG00000125730.18 | C3 | Microglia |
| ENSG00000107099.18 | DOCK8 | Microglia |
| ENSG00000168314.19 | MOBP | Oligodendrocytes |
| ENSG00000197971.16 | MBP | Oligodendrocytes |
| ENSG00000123560.14 | PLP1 | Oligodendrocytes |
| ENSG00000134853.12 | PDGFRA | OPCs |
| ENSG00000173546.7 | CSPG4 | OPCs |
| ENSG00000038427.16 | VCAN | OPCs |
| ENSG00000184113.10 | CLDN5 | Endothelial cells |
| ENSG00000261371.6 | PECAM1 | Endothelial cells |
| ENSG00000169908.12 | TM4SF1 | Endothelial cells |
| ENSG00000102755.12 | FLT1 | Endothelial cells |
| ENSG00000179776.19 | CDH5 | Endothelial cells |
| ENSG00000166825.15 | ANPEP | Pericytes |

Supplementary Table 3 marker genes for Arabidopsis leaf

| geneid | marker | celltype |
|---|---|---|
| AT1G77990 | SULTR2;2 | Bundle sheath |
| AT3G04520 | THA2 | Bundle sheath |
| AT5G57350 | AHA3 | Companion cell |
| AT1G79430 | APL | Companion cell |
| AT1G22710 | SUC2 | Companion cell |
| AT3G22231 | PCC1 | Defense/Salicylic acid |
| AT2G14560 | LURP1 | Defense/Salicylic acid |
| AT5G03350 | AT5G03350 | Defense/Salicylic acid |
| AT1G76540 | CDKB2-1 | Dividing cells: G2/M-phase |
| AT1G44110 | CYCA1-1 | Dividing cells: G2/M-phase |
| AT2G28740 | HIS4 | Dividing cells: S-phase |
| AT3G27060 | TSO2 | Dividing cells: S-phase |
| AT1G07370 | PCNA1 | Dividing cells: S-phase |
| AT1G27950 | LTPG1 | Epidermis |
| AT4G21750 | ATML1 | Epidermis |
| AT1G01120 | KCS1 | Epidermis |
| AT1G54040 | ESP | Epidermis |
| AT3G24140 | FMA | Guard cell/myosin idioblast |
| AT5G26000 | TGG1 | Guard cell/myosin idioblast |
| AT5G25980 | TGG2 | Guard cell/myosin idioblast |
| AT1G08810 | MYB60 | Guard cell/myosin idioblast |
| AT3G16660 | AT3G16660 | Hydathode |
| AT3G16670 | AT3G16670 | Hydathode |
| AT3G14210 | ESM1 | Mesophyll |
| AT1G70760 | NdhL | Mesophyll |
| AT4G12970 | EPFL9 | Mesophyll |
| AT3G27690 | LHCB2.4 | Mesophyll |
| AT3G48740 | SWEET11 | Phloem parenchyma |
| AT5G23660 | SWEET12 | Phloem parenchyma |
| AT5G61480 | PXY | Procambium |
| AT3G15990 | SULT3 4 | Procambium |
| AT2G36120 | DOT1 | Procambium |
| AT3G01680 | SEOR1 | Sieve element |
| AT3G01670 | SEOR2 | Sieve element |
| AT5G19530 | ACL5 | Xylem parenchyma |
| AT4G32880 | ATHB-8 | Xylem parenchyma |
| AT5G60490 | FLA12 | Xylem parenchyma |

# Supplementary Figures

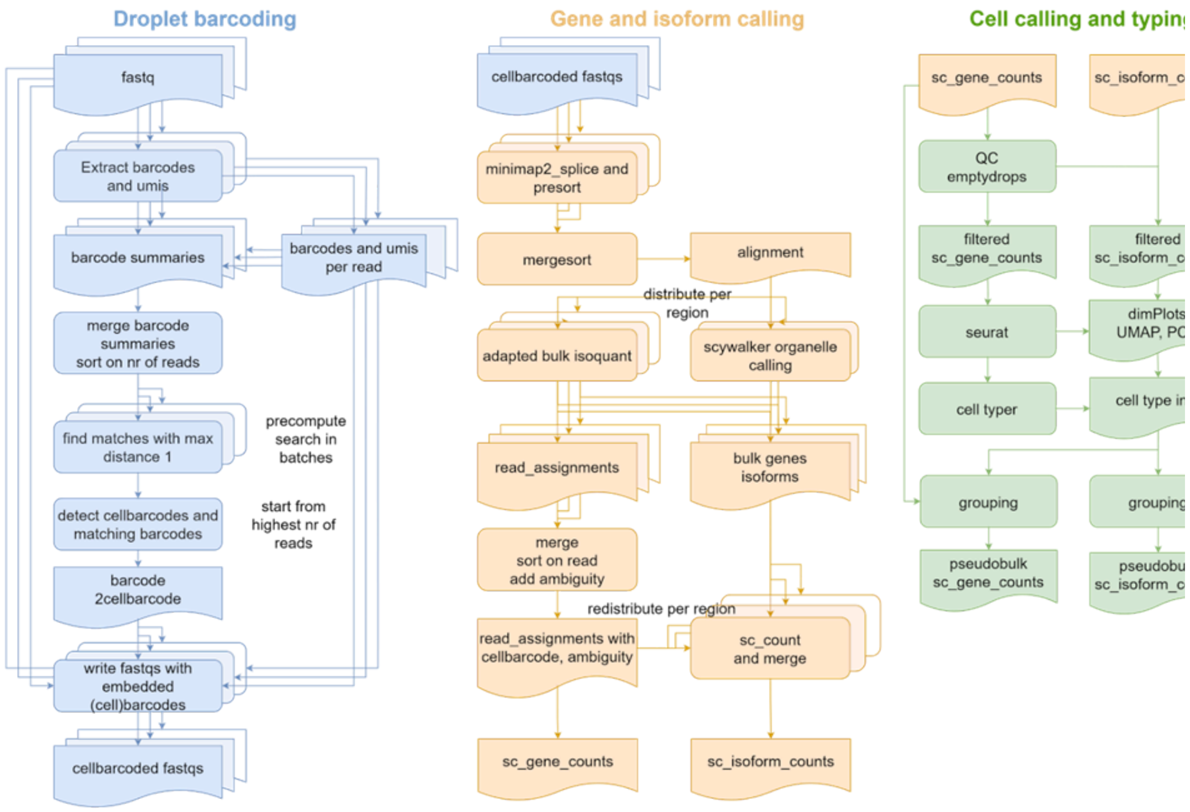


Supplementary Fig 1: Overview of the scywalker workflow. Stacked text boxes indicate parallelized parts. This is the workflow for one sample; multiple samples are always run fully in parallel.

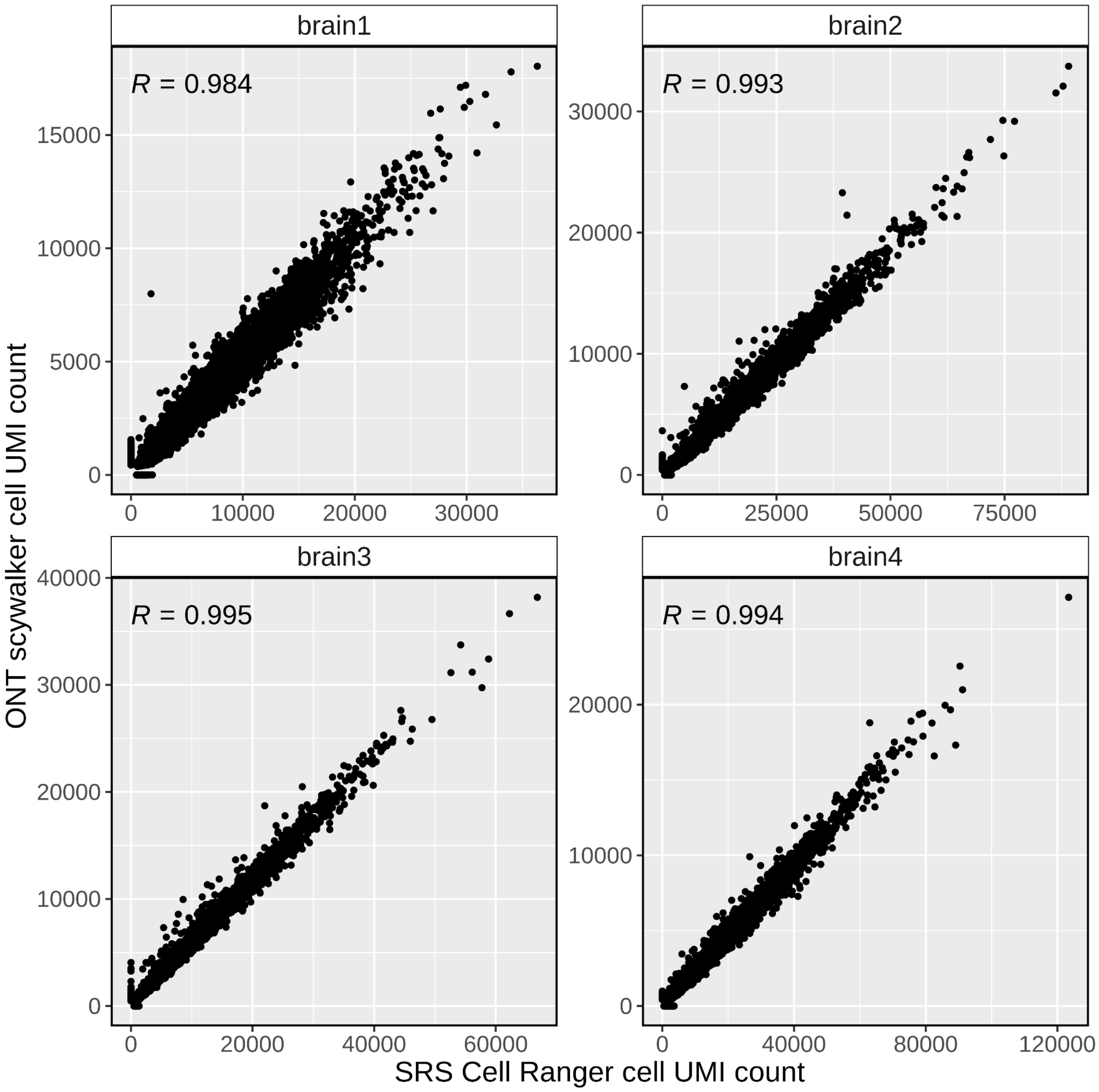


Supplementary Fig. 2: Scywalker UMI counts per cell compared to their respective short-read Cell Ranger results for the four human brain samples. Sample-specific Pearson correlation coefficients (R) are shown on the upper left corners of each panel. *y-axis*, scywalker UMI counts per cell based on long-read sequencing data; *x-axis*, Cell Ranger UMI counts per cell based on short-read sequencing data. SRS, short-read sequencing; UMI, unique molecular identifier.

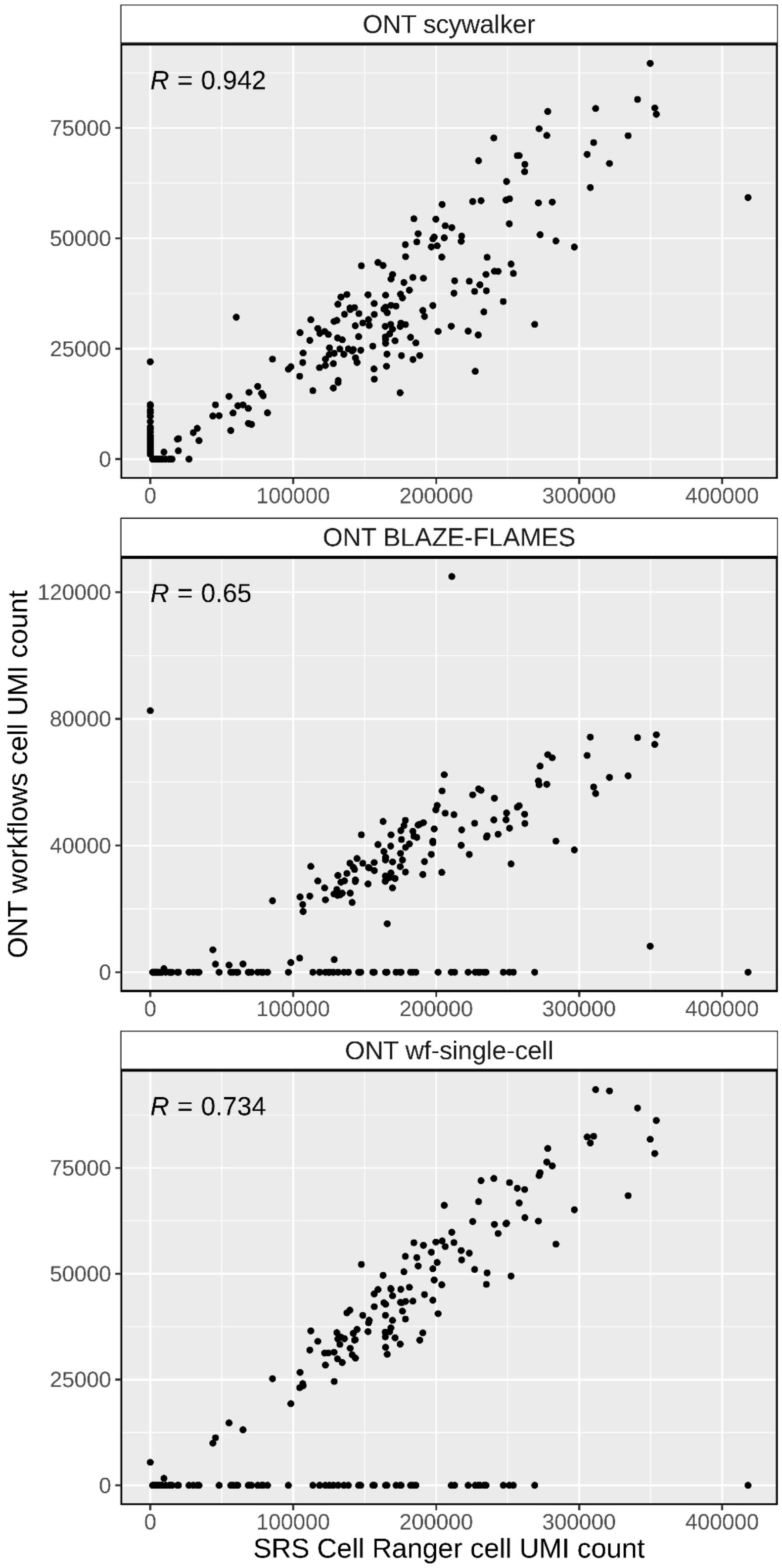


Supplementary Fig. 3: ONT scywalker, BLAZE-FLAMES, and wf-single-cell pipelines derived UMI counts per cell compared to their respective short read Cell Ranger results for the scmixology2 data set. Workflow-specific Pearson correlation coefficients (R) are shown on the upper left corners of each panel. *y* axis, ONT pipeline UMI counts per cell based on long-read sequencing data; *x* axis, Cell Ranger UMI counts per cell based on short-read sequencing data. SRS, short-read sequencing; UMI, unique molecular identifier.

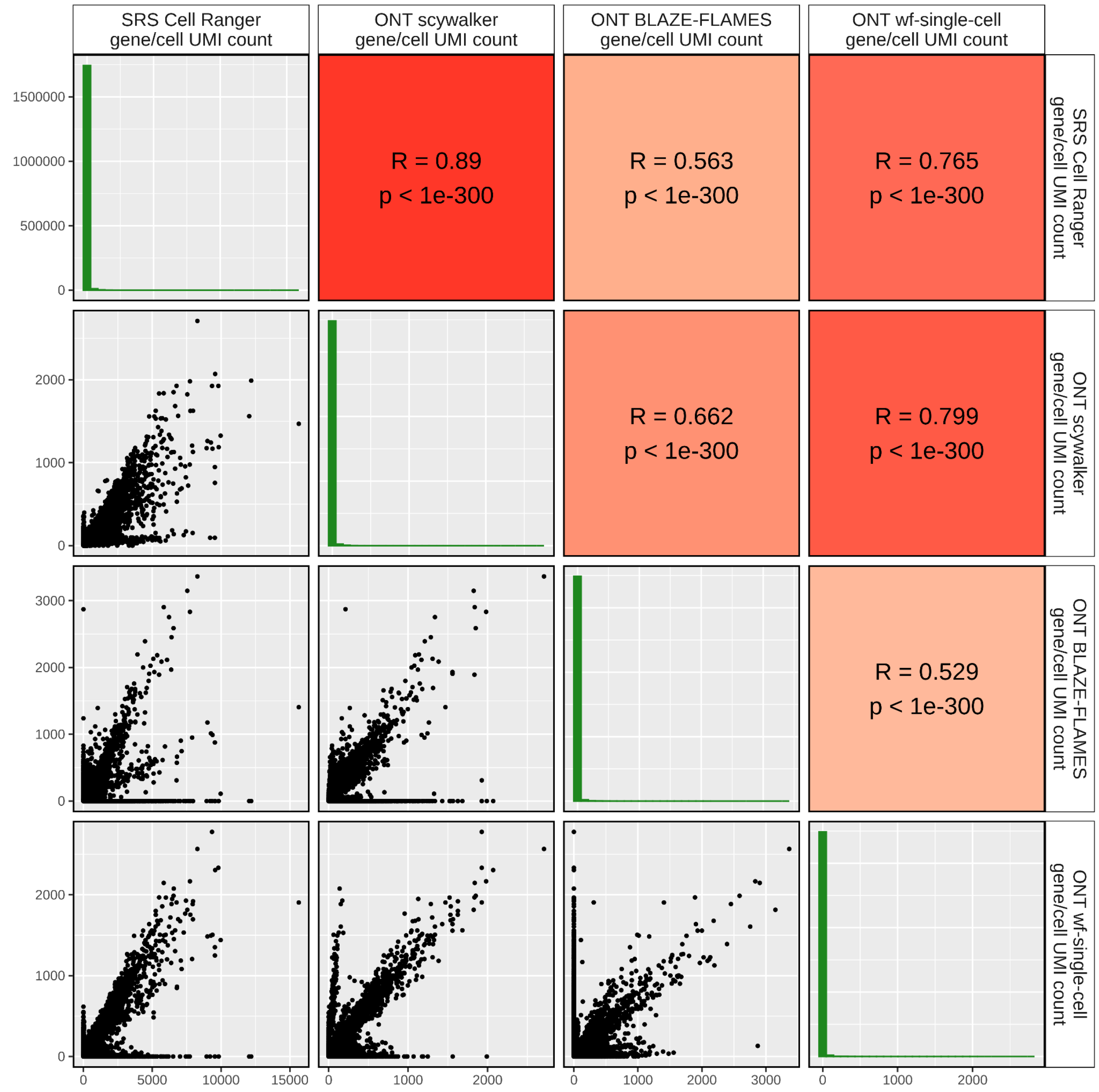


Supplementary Fig. 4: The pairs comparison plot of the UMI counts per gene and cell on the scmixology2 data set using different short and long-read analysis pipelines. The lower part of the matrix shows the scatterplots of the UMI counts per gene and cell derived from different pipelines, the diagonal part of the matrix shows the green histogram bins (n=30) of these counts, while the upper part of the matrix shows the Pearson correlation coefficients (R) and p-values (p) of correlations. A color gradient is used for the upper part of the matrix based on correlation coefficients, where higher coefficients are displayed in a darker red color. SRS, short-read sequencing; UMI, unique molecular identifier.

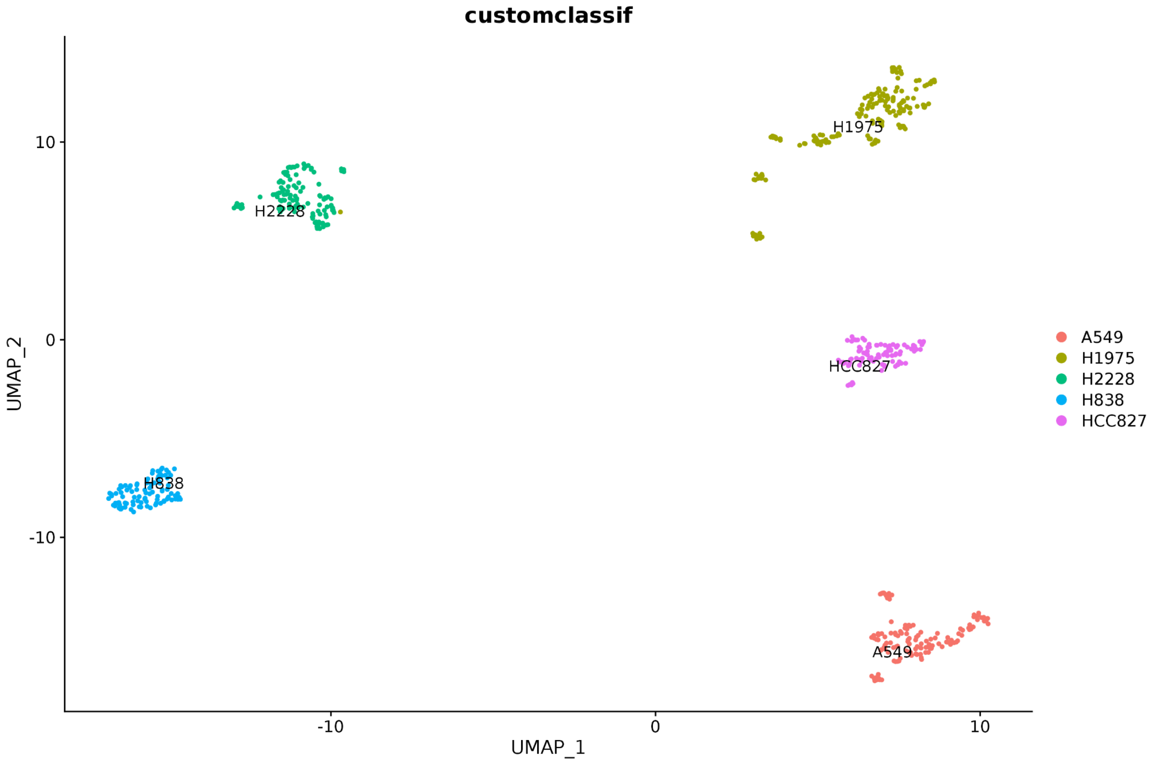


Supplementary Fig. 5: UMAP plot of scywalker based on gene counts in the scmixology2 data set. Cell types predicted by ScType using a custom marker set for this experiment are indicated in color

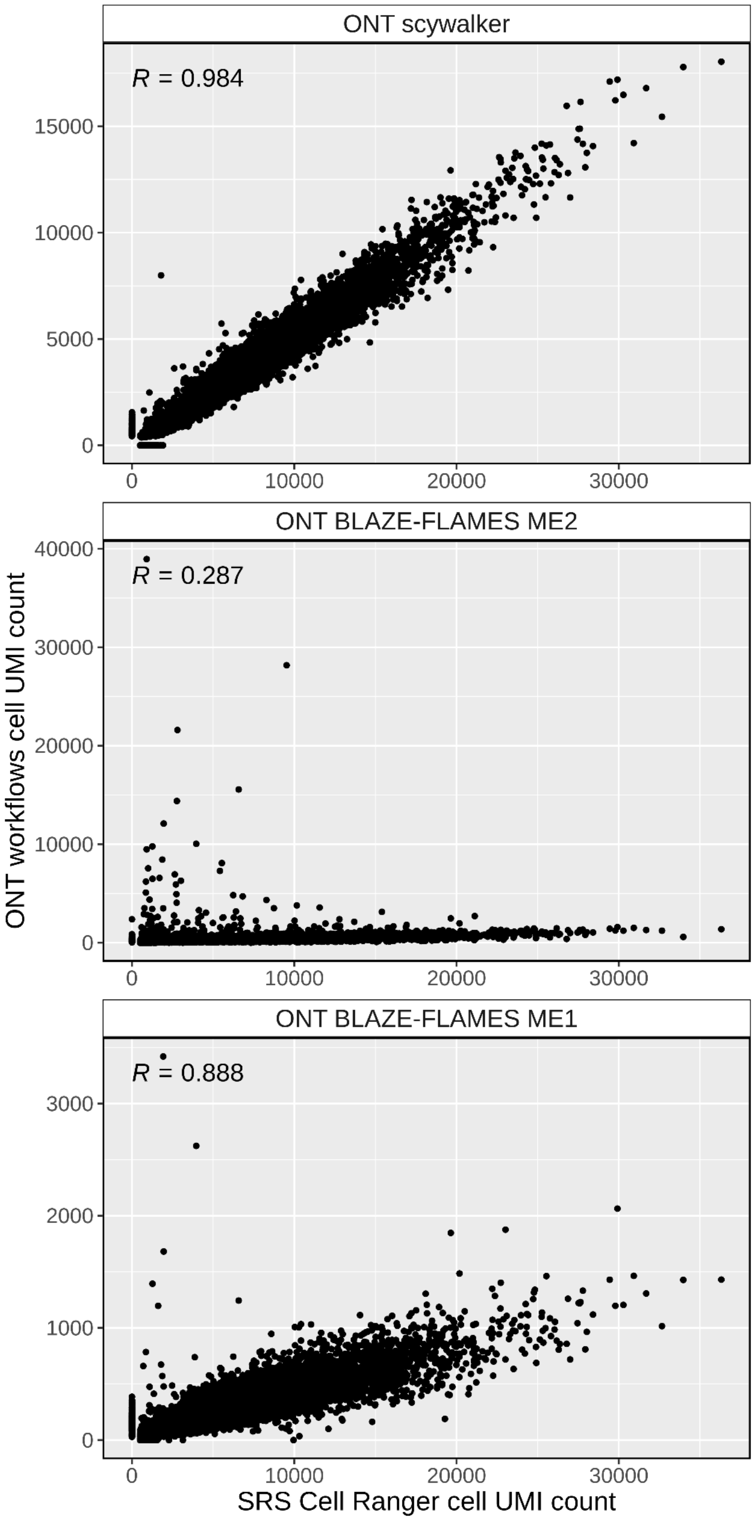


Supplementary Fig. 6: ONT scywalker and BLAZE-FLAMES pipelines derived UMI counts per cell compared to their respective short read Cell Ranger results for the brain1 data set. BLAZE-FLAMES pipeline was run with both max edit distance settings of 2 (ME2) and 1 (ME1). Workflow-specific Pearson correlation coefficients (R) are shown on the upper left corners of each panel. *y-axis*, ONT pipeline UMI counts per cell based on long-read sequencing data; *x*-axis, Cell Ranger UMI counts per cell based on short-read sequencing data. SRS, short-read sequencing; UMI, unique molecular identifier; ME, max edit.

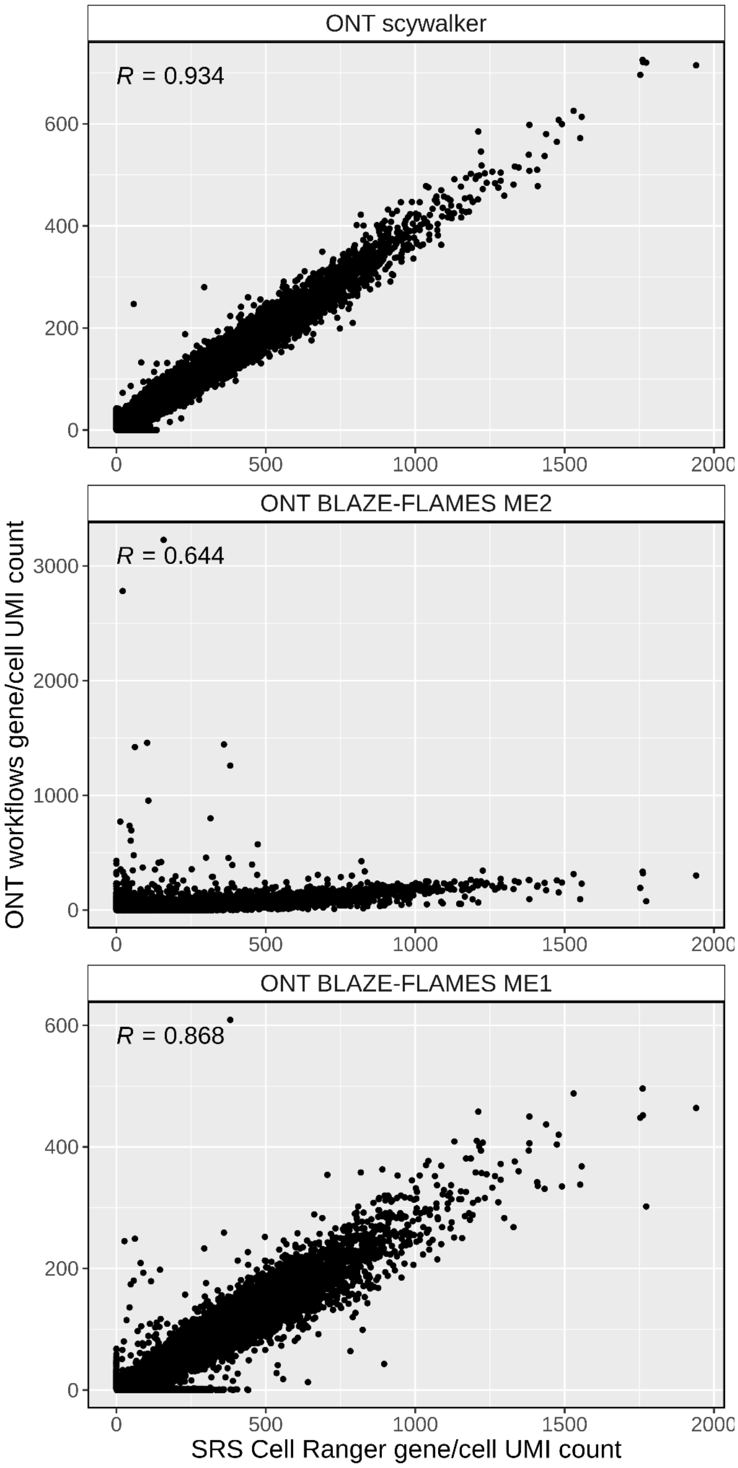


Supplementary Fig. 7: ONT scywalker and BLAZE-FLAMES pipelines derived UMI counts per gene and cell compared to their respective short read Cell Ranger results for the brain1 data set. BLAZE-FLAMES pipeline was run with both max edit distance settings of 2 (ME2) and 1 (ME1). Workflow-specific Pearson correlation coefficients (R) are shown on the upper left corners of each panel. *y-axis*, ONT pipeline UMI counts per gene and cell based on long-read sequencing data; *x-axis*, Cell Ranger UMI counts per gene and cell based on short-read sequencing data. SRS, short-read sequencing; UMI, unique molecular identifier; ME, max edit.

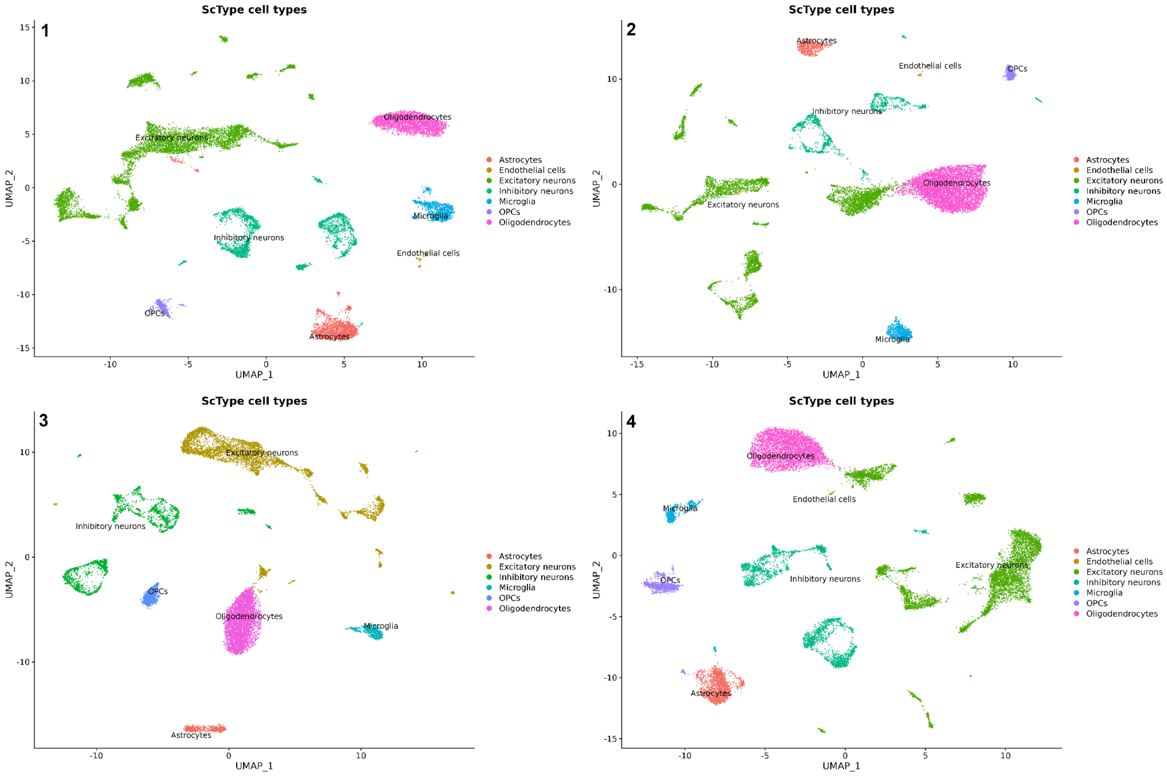


Supplementary Fig. 8: UMAP plots generated by scywalker showing cell-type assignments by ScType in different colors for datasets brain 1-4.

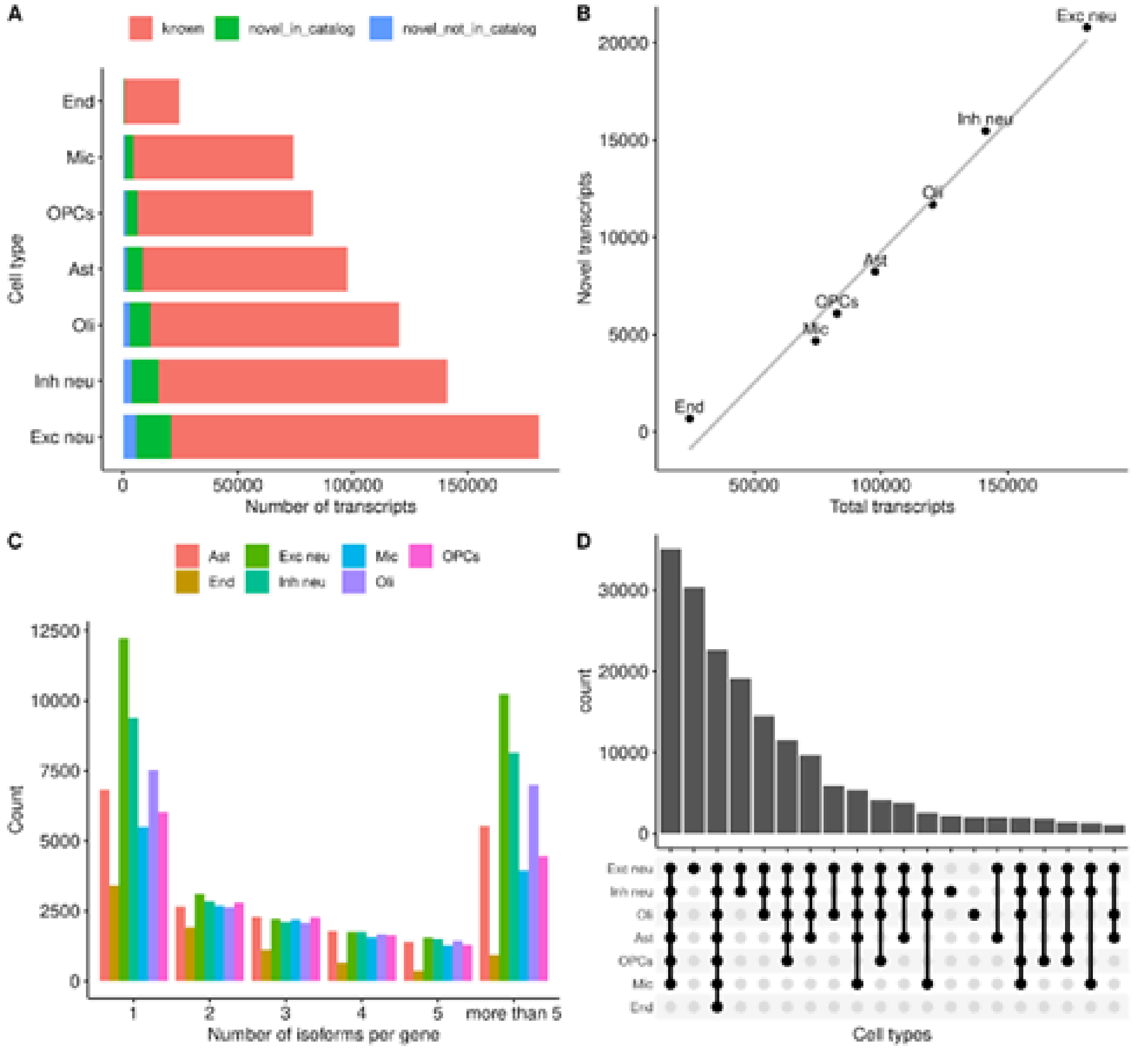


Supplementary Fig. 9 Overview of the scywalker performance at the isoform level. (A) Bar plot representing the number of transcripts identified by cell type. Colors represent different transcript types. (B) Scatter plot correlating the number of total transcripts and novel transcripts (Pearson correlation R=0.99, p=1.9x10$^{-5}$). (C) Bar plot showing the number of isoforms per gene by cell type. (D) Upset plot representing the intersections of identified transcripts per cell type. Exc neu: excitatory neurons; Inh neu: inhibitory neurons; Ast: astrocytes; End: endothelial cells; Oli: oligodendrocytes; OPCs: oligodendrocyte progenitor cells.

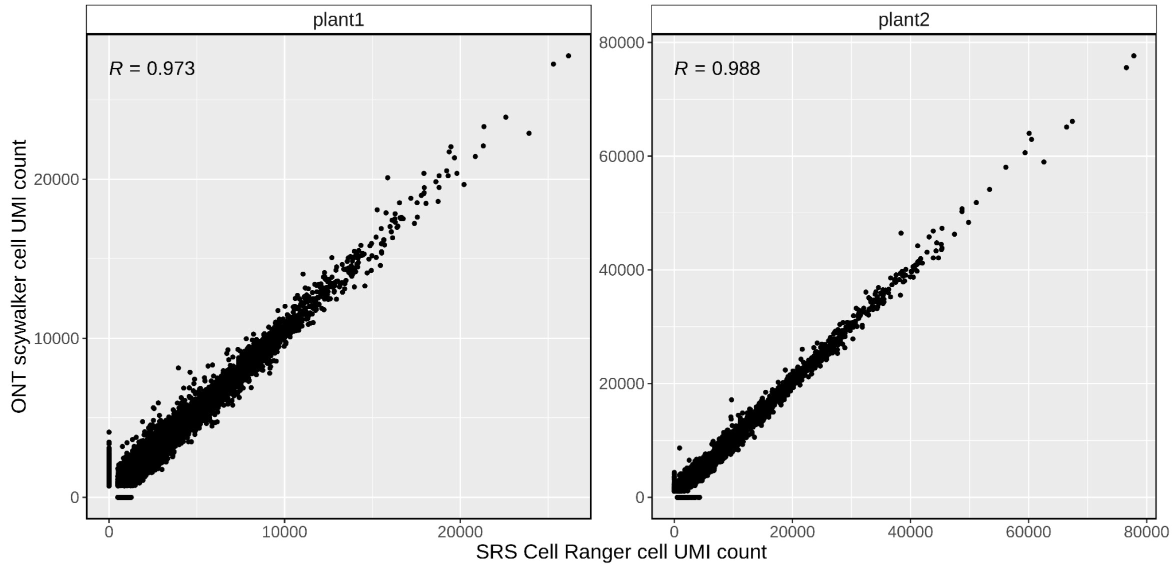


Supplementary Fig. 10: Scywalker UMI counts per cell compared to their respective short-read Cell Ranger results for the two plant samples. Sample-specific Pearson correlation coefficients (R) are shown on the upper left corners of each panel. *y-axis*, scywalker UMI counts per cell based on long-read sequencing data; *x-axis*, Cell Ranger UMI counts per cell based on short-read sequencing data. SRS, short-read sequencing; UMI, unique molecular identifier.

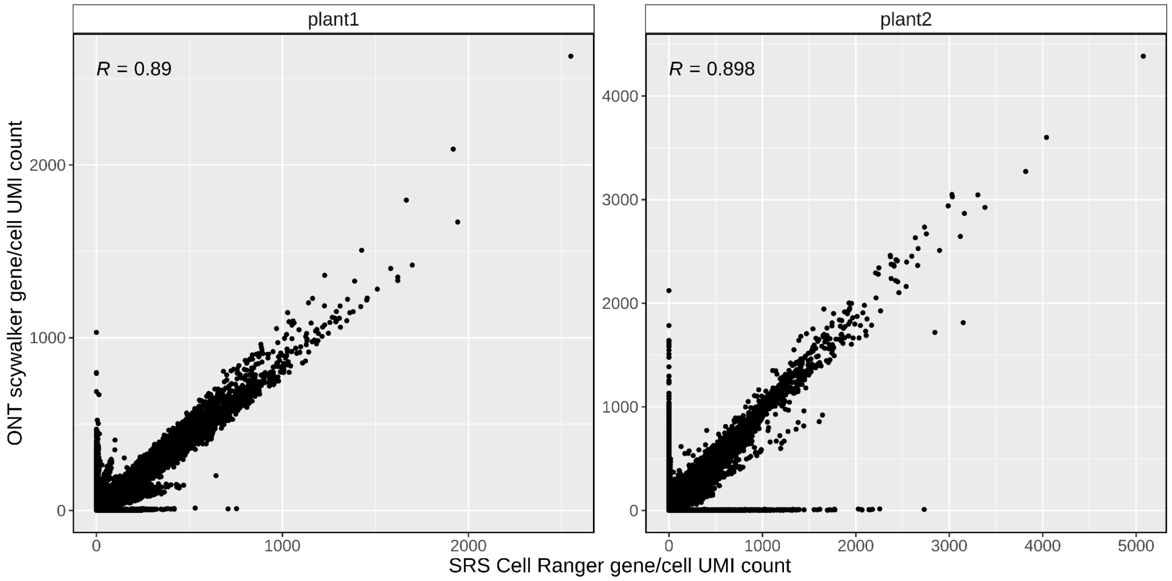


Supplementary Fig. 11: Scywalker UMI counts per gene and cell compared to their respective short-read Cell Ranger results for the two plant samples. Sample-specific Pearson correlation coefficients (R) are shown on the upper left corners of each panel. *y-axis*, scywalker UMI counts per gene and cell-based on long-read sequencing data; *x-axis*, Cell Ranger UMI counts per gene and cell-based on short-read sequencing data. SRS, short-read sequencing; UMI, unique molecular identifier.

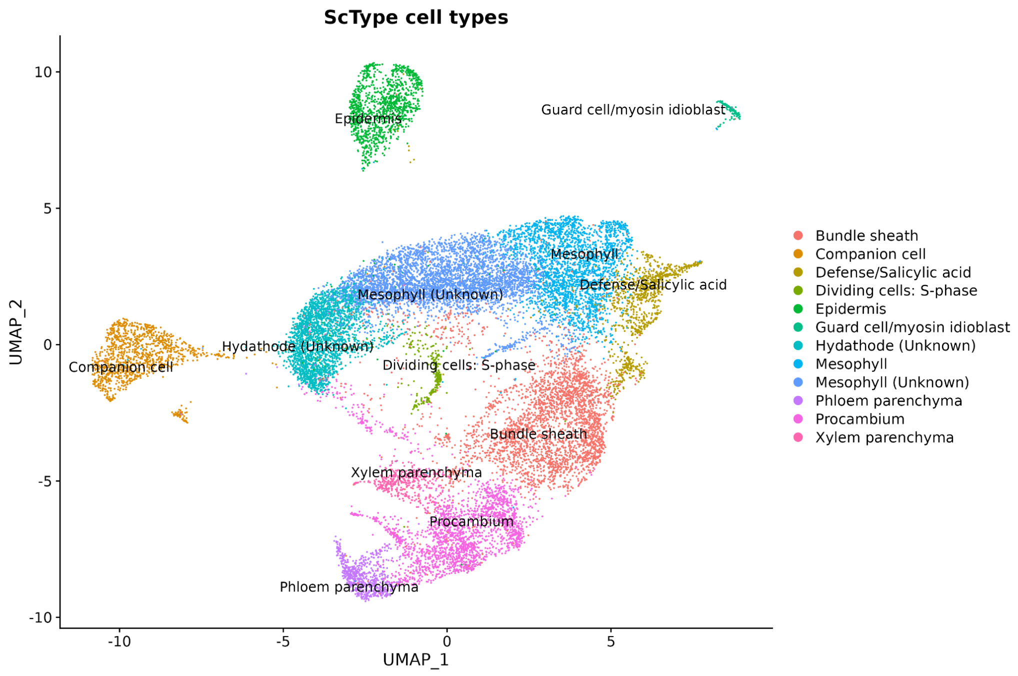


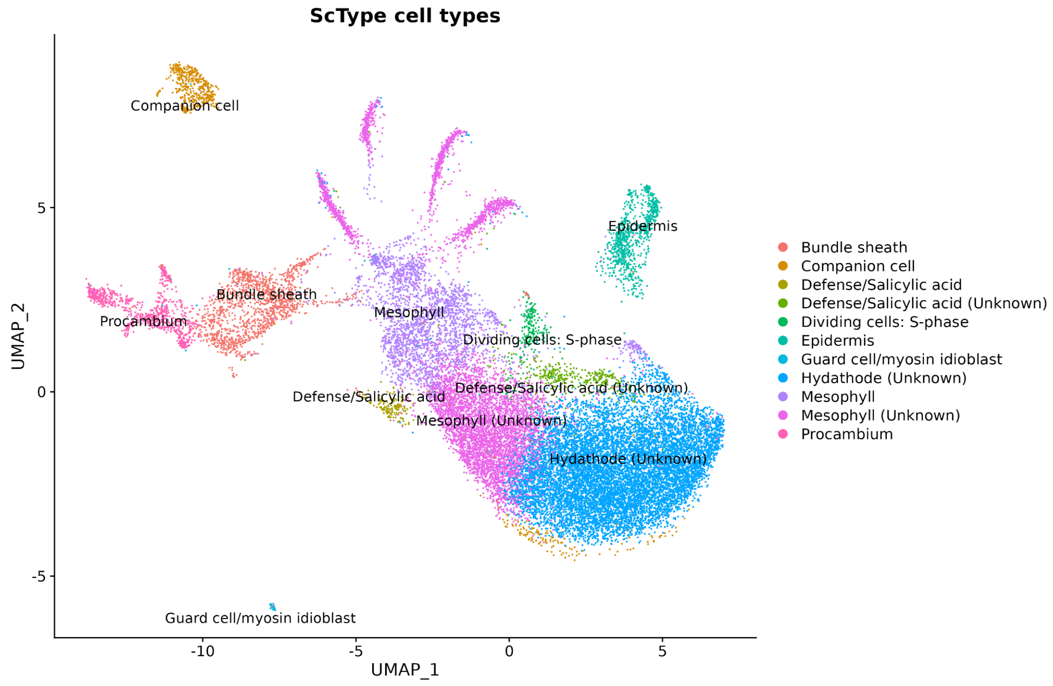


Supplementary Fig. 12: UMAP plot generated by scywalker showing cell-type assignments by ScType in different colors for plant sample 1 and 2.

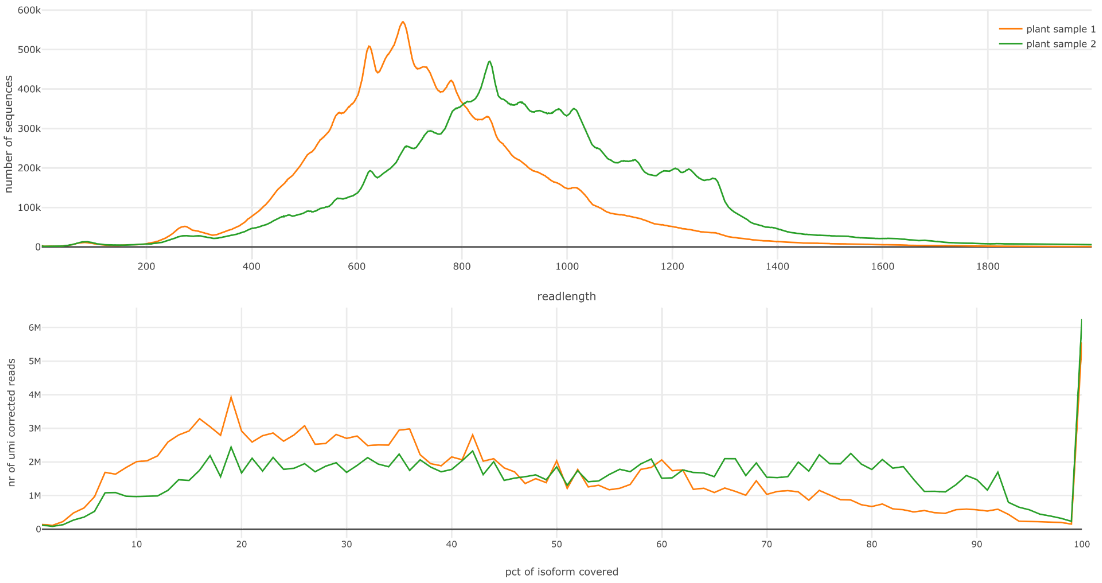


Supplementary Fig. 13 Effect of read length on coverage. The top panel shows the distribution of read lengths for plant samples 1 and 2. The bottom panel shows the number of reads covering a certain percentage of their isoform.

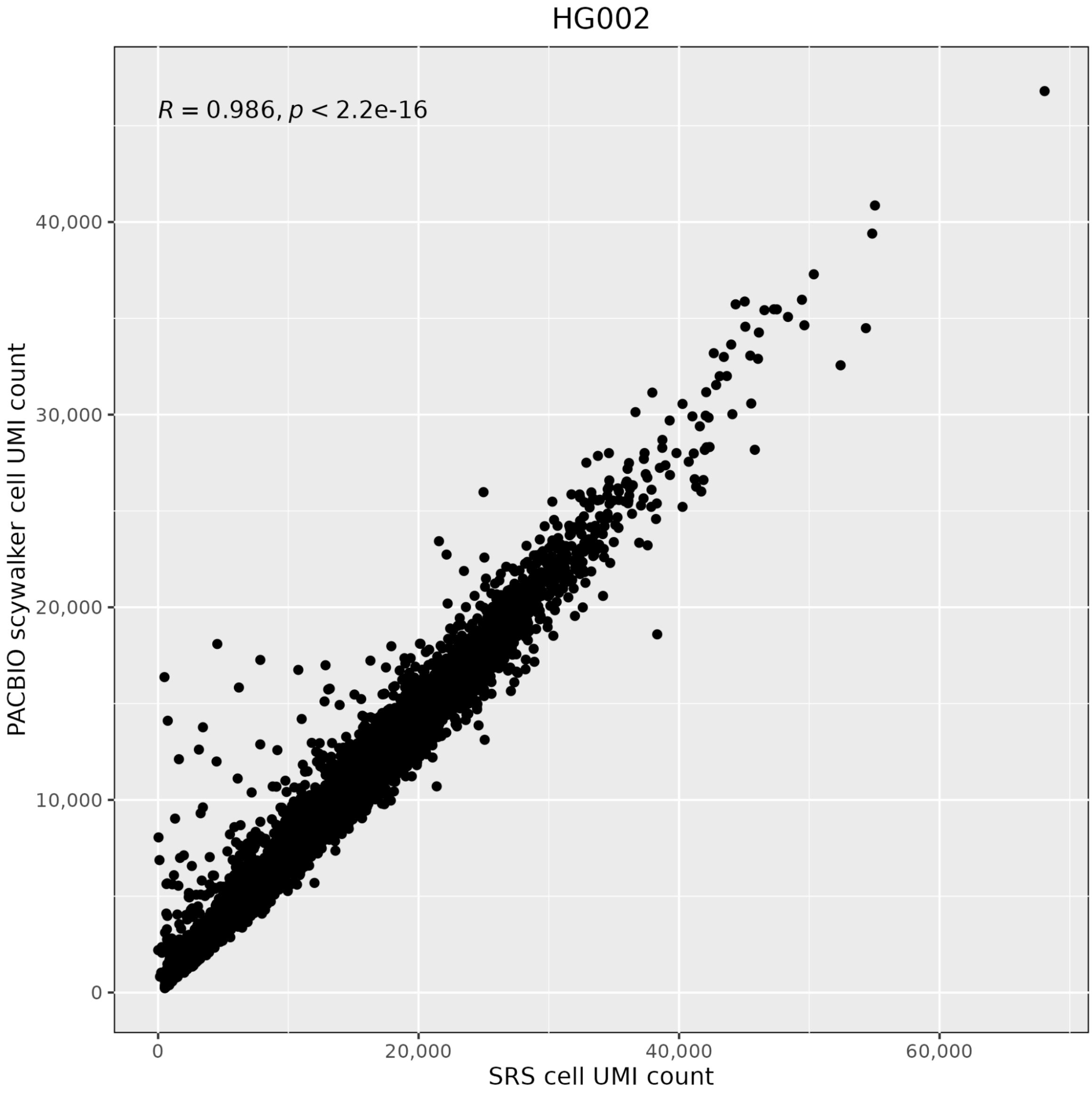


Supplementary Fig. 14: Scywalker UMI counts per cell compared to their respective short read Cell Ranger results for PacBio HG002 data. Sample-specific Pearson correlation coefficients (R) are shown on the upper left corners of each panel. *y-axis*, scywalker UMI counts per cell based on long-read sequencing data; *x-axis*, Cell Ranger UMI counts per cell based on short-read sequencing data. SRS, short-read sequencing; UMI, unique molecular identifier.

test

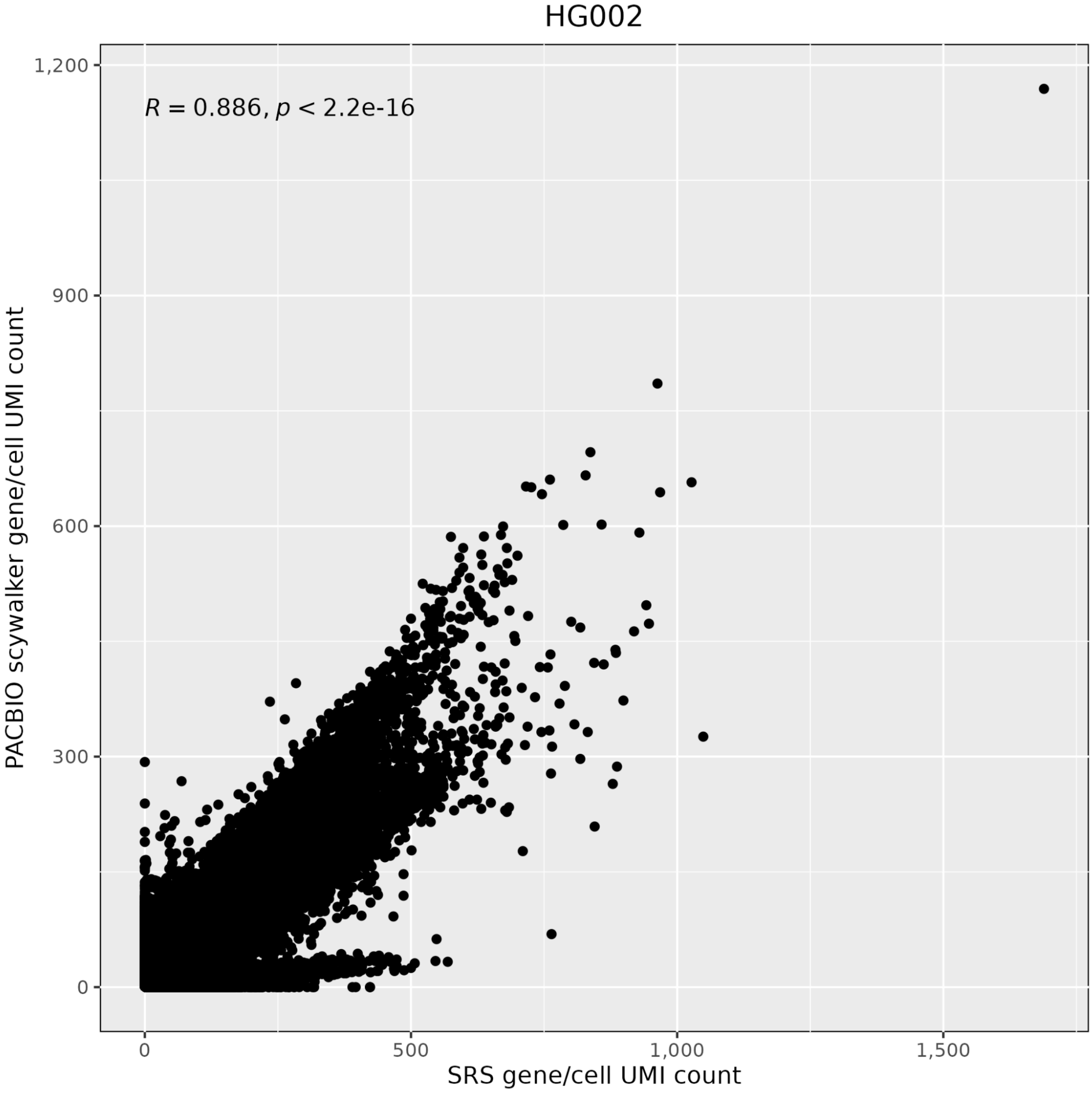


Supplementary Fig. 15: Scywalker UMI counts per gene and cell compared to the corresponding short-read Cell Ranger results for PacBio HG002 data. For this comparison, reads assigned to immunoglobulin (IG) “genes” were filtered out because, in the reference, all potential gene segments are indicated as separate genes: As long reads determine the full transcripts (combining the rearranged segments), the individual segments are not counted as in the short read data.
Sample-specific Pearson correlation coefficients (R) are shown on the upper left corners of each panel. *y-axis*, scywalker UMI counts per gene and cell based on long-read sequencing data; *x-axis*, Cell Ranger UMI counts per gene and cell based on short-read sequencing data. SRS, short-read sequencing; UMI, unique molecular identifier.

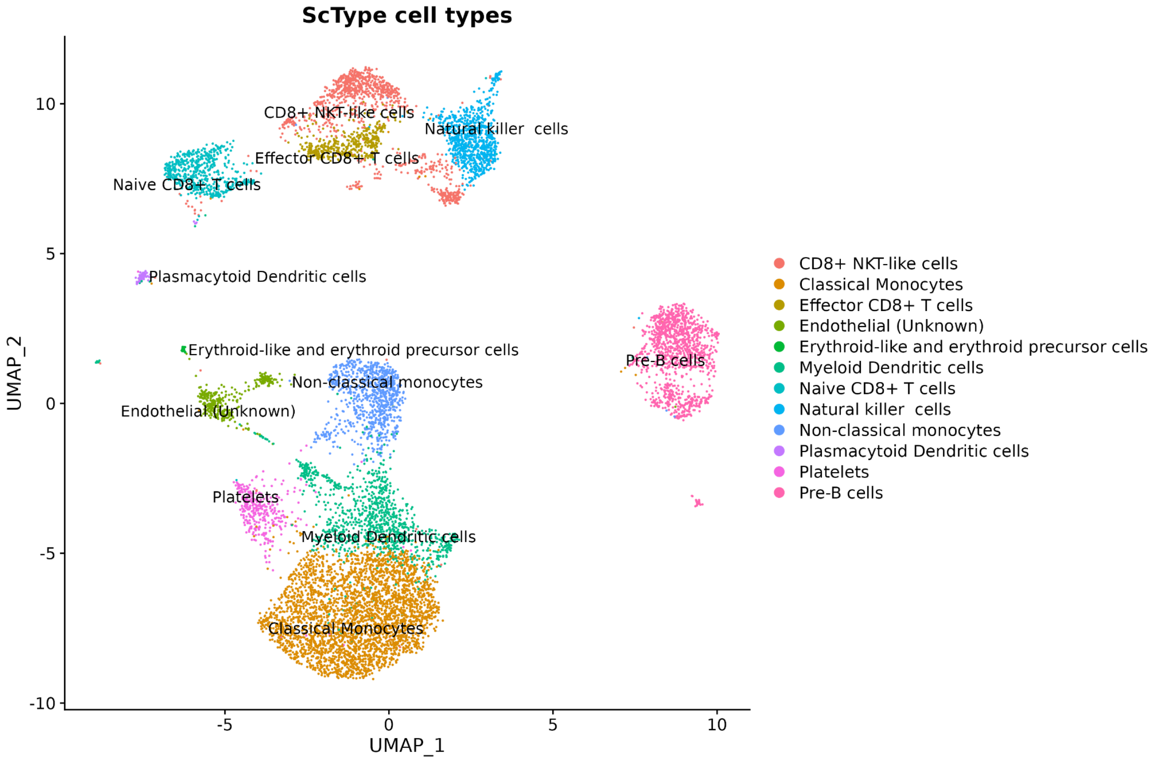


Supplementary Fig. 16: UMAP plot generated by scywalker based on gene counts showing cell-type assignments by ScType in different colors for the PacBio PBMC sample.

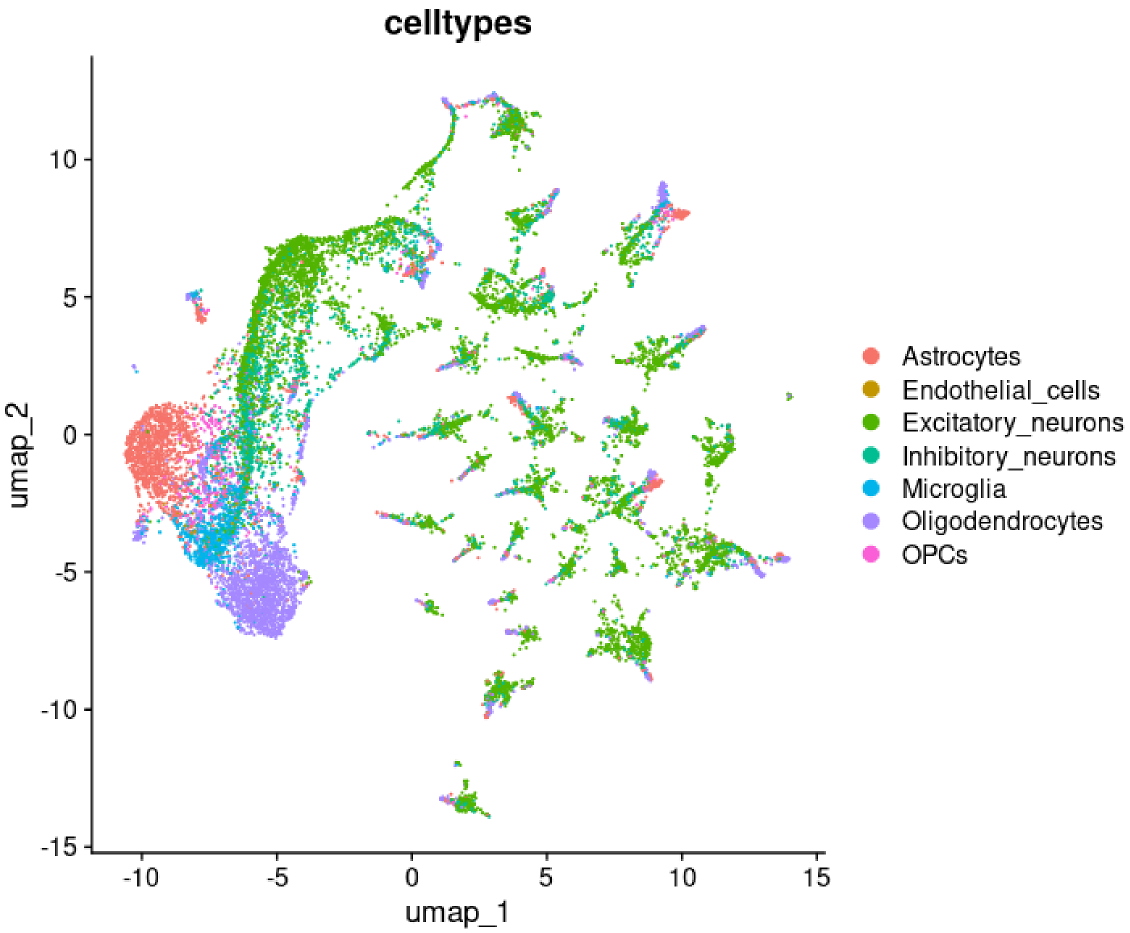


Supplementary Fig. 17: UMAP plot generated by clustering based on transcript counts, showing cell-type assignments obtained by ScType using scywalker gene counts.
